# A new paradigm for the scientific enterprise: nurturing the ecosystem

**DOI:** 10.12688/f1000research.15078.1

**Published:** 2018-06-20

**Authors:** Alexander K. Lancaster, Anne E. Thessen, Arika Virapongse

**Affiliations:** 1 Ronin Institute for Independent Scholarship, Montclair, New Jersey, USA; 2 Amber Biology, Cambridge, Massachusetts, USA; 3 The Data Detektiv, Waltham, Massachusetts, USA; 4 Middle Path EcoSolutions, Boulder, Colorado, USA

**Keywords:** academia, higher education, independent scholarship, careers, science studies, politics of science, systems-thinking, peer-to-peer science, collaboration

## Abstract

The institutions of science are in a state of flux. Declining public funding for basic science, the increasingly corporatized administration of universities, increasing “adjunctification” of the professoriate and poor academic career prospects for postdoctoral scientists indicate a significant mismatch between the reality of the market economy and expectations in higher education for science. Solutions to these issues typically revolve around the idea of fixing the career "pipeline", which is envisioned as being a pathway from higher-education training to a coveted permanent position, and then up a career ladder until retirement. In this paper, we propose and describe the term “ecosystem” as a more appropriate way to conceptualize today’s scientific training and the professional landscape of the scientific enterprise. First, we highlight the issues around the concept of “fixing the pipeline”. Then, we articulate our ecosystem metaphor by describing a series of concrete design patterns that draw on peer-to-peer, decentralized, cooperative, and commons-based approaches for creating a new dynamic scientific enterprise.

## Introduction

The institutions of science are in a state of flux. Declining public funding for basic science ^1, 2^ has led academic institutions to change their business models ^3, 4^; the administration of universities is becoming increasingly corporatized ^5^. With increasing “adjunctification” of the professoriate ^6^, continued use of outdated funding models for research science in academia, and dwindling academic career prospects for postdoctoral scientists ^7– 10^, it is clear that there is a mismatch between the reality of the market economy and expectations in higher education. 

The evolving funding landscape at academic and research institutions has had a major impact on career opportunities for scientists, particularly those who are early-career. As a result of grant dollars being increasingly awarded to a disproportionately small number of established investigators and institutes ^11^, intellectual discovery has become captured by a privileged few ^12^, leading to greater bias in scientific research, diminished scientific productivity ^13^, and less potential for breakthrough discoveries ^14, 15^. Such a lack of social diversity and equity is a major challenge in science, technology, engineering, and mathematics (STEM) ^16, 17^. Solutions are often sought out by proposing adjustments to the “career pipeline”, but these issues in STEM continue to be unresolved.

**Figure 1.**
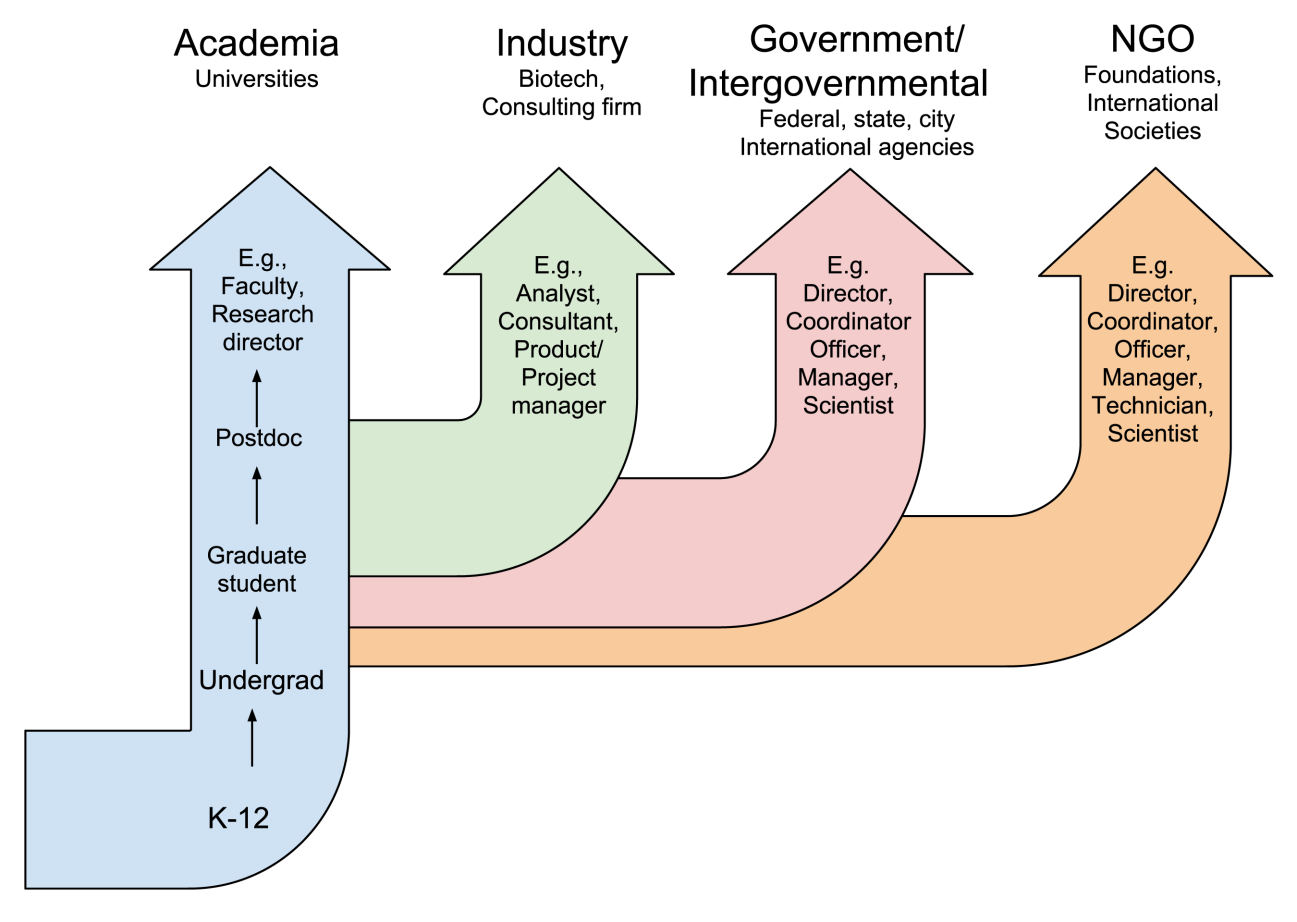
The standard scientific career “pipeline”. The pipeline includes formal scientific training and different scientific career paths. The pipeline is characterized as a set of distinct streams with little flow between each stream, and a career ladder within each sector.

The career pipeline envisions a straight career path, from higher-education training to a coveted permanent position, and then up a career ladder until retirement ( Figure 1). While such a direct path to success may be optimal for some, it does not reflect the reality of typical career development ( Box 1). In today’s economy, permanent positions are becoming rarer across all industries, including universities, which are as more contract positions that are short-term and require no employee benefits are offered ^18^.

Box 1. **The PhD “path” to a tenure-track position**

The alarming disproportion between the number of people with PhDs and the number of university-based academic positions available for PhDs has become a major preoccupation in the trade science press and beyond ^25^. Indeed, training models for graduate students, and particularly for PhDs, in STEM often focus on delivering them to a tenure-track faculty position. Decades-long reliance on graduate students and post-doctoral researchers as cheap labor contributes to today’s unsustainable academic models and underemployment of academic scientists. Such a system has become a poverty trap for many graduate students and early PhDs, as they work long hours for low wages under the expectation that their participation in the pipeline will eventually lead to a permanent position in academia. The reality is that only 8% of postdocs are able to land a tenure-track job within 6 years of being awarded a PhD ^26^.

Much of the discussion around career prospects for PhDs assumes that they must find a traditional position in a university in order to continue pursuing their scientific goals ^27^. Funding changes have also produced an academic structure that provides limited prospects for early-career scientists to advance their careers within academia ^9^. Postdoctoral training periods also continue to expand. While the increasing complexity of research may require longer training periods, it is unlikely that longer postdoc positions result in better researchers; many postdocs rarely get the appropriate direct training and mentoring to start an independent lab ^28^. It is unknown how many promising, early-career scientists become trapped in postdoctoral limbo, as the morass of titles given to postdocs disguises the scale of the scientific workforce that exists in this state ^29^. The ever-lengthening apprentice time for scientists has created a Red Queen's Race: scientists must run twice as fast to stay in the same place with their point of “independence” postponed almost indefinitely ^30^.

Additionally, an outdated mentality still persists that the path to faculty tenureship requires putting science ahead of all of life’s other priorities, and this can have a severely negative effect on the mental health of those who try to conform (e.g., 31). While this model may have worked decades ago for those (mostly male) scientists that could rely on compliant spouses to raise families and perform domestic duties, it does not work in today's world. By presenting it as such, the pool of tenured faculty is limited to those who have the means to commit to such a lifestyle: typically young, male, unencumbered with children and geographically unconstrained. This demographic is steadily decreasing proportionally across the whole scientific research community, so career advice solely targeting this group is increasingly irrelevant.

Even after gaining a tenure-track position, the mechanics of gaining tenure can be just as rigid and unforgiving ^32^. The intense pressure on faculty to be “productive” (i.e., larger grants, publications in “high impact” journals), as well as the race to achieve institutional measures of “excellence” ^33^, all divert attention from scholarship ^2, 28, 34, 35^ and undermine the creativity that nourishes scientific progress ^36– 38^. There have been serious concerns that such hypercompetition has led to a drop in scientific quality, especially in the biomedical field, as labs vie to publish their papers in the limited number of “slots” in high profile journals ^39, 40^. Indeed, the existence of fairly accurate predictive models ^41^ of one’s chances of “becoming a principal investigator” based on affiliation, publication, and grant metrics, is itself a symptom of a profoundly narrow view of “success” in science.

The limitations of the pipeline as a conceptual model for education and careers is being recognized in both the tech industry ^19^ and science ^20^. The consequences of continuing to apply this outdated model is stalled career development in science, underemployment of some of the most highly educated people in our society, and overall loss of STEM professionals as they seek out career alternatives ^21– 23^. In 2016 $3 billion were invested into federal agencies to support STEM education programs ^24^. Considering the governmental and individual investment that is made into higher STEM education each year, this is not just an academic conundrum—it is a societal problem.

By persisting with the assumption of the pipeline, we also miss engaging in conversations that address the fundamental cultural change that is occurring in science today. A new conceptual model is needed to help guide both early-career scientists and those who care about the scientific enterprise towards a more sustainable and resilient professional future. In this paper, we propose and describe the term “ecosystem” as a more appropriate way to conceptualize today’s scientific training and the professional landscape of the scientific enterprise. First, we highlight the issues around the concept of “fixing the pipeline”. Then, we articulate our ecosystem metaphor by describing a series of design patterns that draw on peer-to-peer, decentralized, cooperative, and commons-based approaches to science. We finish by describing the related cultural shifts underway that can hasten a more diverse and fluid scientific enterprise into the 21st century.

## Fixing the pipeline

Much effort has gone into improving the efficiency of the scientific “career pipeline” ( Figure 1). Proposed solutions tend to fall into one of three categories: adjusting the rates of flow in the pipeline, modifying oneself to fit the pipeline, or switching to an “alternative” pipeline. These solutions focus primarily on accessing tenure track faculty positions, as this issue has received the most attention in literature regarding the challenges facing the “pipeline” for scientists ( Box 1). Here, we highlight some of the main issues facing these proposed solutions. 

### Adjusting flow in the pipeline

Alberts *et al.* ^35^ proposed that the supply of the pipeline (i.e., the number of trainees) should be adjusted to match the demand (i.e., the number of tenure-track positions). Increasing the number of tenure-track-style positions, as desirable as that might be, seems unlikely given the current trends in science funding. By addressing the demand end, it has also been suggested that the size of individual labs should be decreased to reduce the number of trainees that must be moved into faculty positions ^10, 42^. While these proposed reforms are thoughtful and well-intentioned, they do not address the problems of an oversupply of talented researchers and funding models that rely on large numbers of low-paid trainees to get work done. Such a solution could certainly lead to fewer scientists with more career stability, but is this the future that we envision for science and our society?

### Adapting to the pipeline

Today’s search for a tenure track position is often a multi-year process as many open positions are saturated with candidates ^43^. Many candidates must go to great lengths to make themselves more competitive. Recognizing this need, academic departments, as well as for-profit companies ^44^, now assist with academic “survivorship” to help potential candidates prepare CVs, develop interview skills, and write research statements. This is leading to a system that is even less accessible to those with lesser means ^45^ and may contribute even further to a bias towards hiring candidates from elite institutions ^46^. It also contributes to the ever-lengthening time period before a scientist can “fledge” as a professional in academia. Much of the energy, currently being devoted to preparing individuals to adapt to the system, could be redirected towards more collaborative and collective solutions.

### Finding another pipeline

The paucity of traditional academic jobs has led to increased career advice about alternatives to academic careers, and there are encouraging indications that many PhDs are finding employment outside academia ^47, 48^. Although most of these efforts are entirely well-meaning, they sometimes have the unintended consequence of reinforcing the same tendencies as “pipeline-thinking”. For example, a recent *Nature* editorial discussed the ability of PhDs or postdocs to “stay in touch with science” by working in a “related function such as administration, outreach or publishing” ^48^. While these career options suit many people, the possibility of doing any future self-directed science outside of an academic or research position is rarely considered. Such a narrative is disempowering overall, as junior researchers are encouraged to “take a hard look at their job prospects” rather than question the nature of institutions or the pipeline ^25^. Other discussions of the STEM pipeline are framed by discussions of “workforce needs” and the production of workers that are “globally competitive” ^49, 50^, reinforcing a business approach to science ^51^, rather than on doing science as a public good or for its own sake ^52^.

### Thinking outside the pipeline

Much of the career advice on how to be successful in the academic science pipeline reflects the values and dynamics present in the job market when many senior scientists obtained their first positions (e.g., that outlined by Diamandis ^53^), instead of the realities of today ^54, 55^. As many of these scientists’ career experiences are often limited to academia, their perspectives may not be relevant to early-career scientists who are open to other career options. Academics often fail to recognize the broad applicability and value of PhD degrees, or encourage their trainees to work outside the traditional academic pipeline.

By framing solutions in terms of “fixing the pipeline”, the underlying career structures for scientists remain largely unchallenged. As such, early-career scientists occupy a passive role, waiting for change to come from the top, such as through institutional reform driven by senior leaders. Likewise, the scarcity of research positions is accepted as a given, limiting how much science can be done. There is, no limit on society’s need to address complex challenges, the number of research questions that can be asked, or the amount of scientific *work* that can be done. New models are needed to help identify different ways for scientists to continue their work outside of a standard academic or agency job. 

## The science ecosystem

**Figure 2.**
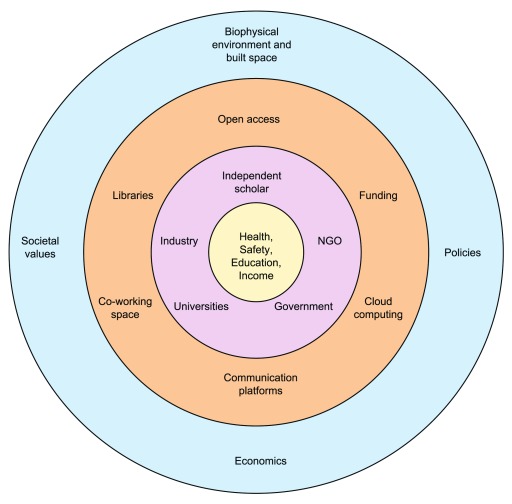
The scientific ecosystem. The inner circle (beige) represents the basic necessities needed to be a functioning member of society, as well as a scientist. The next circle (purple) shows the different groups that are often involved in the pursuit of knowledge and scientific progress. Because the circle can be rotated there is no ‘up’ in this representation; no one type of institution is privileged in this representation and there is exchange in all directions. In addition, the borders between the different institutes are highly porous—there is collaboration, reflection, and sharing of resources between them. The next circle (orange) represents different kinds of resources and infrastructure needed to support science. The outermost circle (light blue) represents the environmental context, including biophysical limitations, and the socio-political and economic landscape, that science and scientists must function within, adapt to, and seek to understand and affect.

We propose an ecosystem as a conceptual model that is relevant both to the training of a scientist and their role as a professional ( Figure 2). The two most inner circles in the Figure depict the basic necessities, training, and professionalism of science. Here, traditional scientific labs may still have a role, but the networks of peer-to-peer collaborators that span both within and outside of institutions are emphasized. The two outermost circles are the impetus behind the changing context of science today. It is becoming more evident that a new systems-based approach is needed to allow science to adapt more quickly to the complex socio-political and biophysical context of today (the outermost circle). There are, however, now new resources, tools, and infrastructure (courtesy of STEM advances), such as lab space, journal access, and high-performance computing, either publicly available, or available for rent, that allow science to thrive outside of traditional institutions (the orange, next outermost circle) ^56^. In addition, bottom-up changes are already being driven by early career scientists themselves in many different ways ^57– 60^.

Many postdocs and adjunct scientists already have the majority of tools that they need to do independent science, such as deep training and understanding of their field, a body of work that demonstrates their scientific ability, pre-existing networks of colleagues with similar intellectual interests, and the Internet to collaborate and share. By moving beyond the existing pipeline model of academic science, the ecosystem vision provides the space, flexibility, and diversity that science needs to be more responsive to both local and broader complex scales affecting science.

To demonstrate how an ecosystem model would work in practice, we present a set of conceptual design patterns loosely inspired by commons-based approaches ^61– 63^, systems-thinking approaches ^64^, and the sustainable livelihoods framework ^65^. We acknowledge specific social movements and grassroots changes that are occurring today, and demonstrate how science now has the means to be more egalitarian, inclusive, and diverse by being less dependent on their institutional settings. We recognize, that major institutional reforms are needed to realize this vision to its fullest, so we also address the changing role of institutions within this vision. We deliberately chose the term “ecosystem” to resonate with many of the phenomena that exist in biological ecosystems: diversity, resilience, multiplicity of scales, dynamic feedback loops, etc., and we use some of these concepts when framing each of the design patterns. That said, we do not claim a one-to-one correspondence with biological ecosystems.

### 1. Fundamental development of the scientist

Basic necessities (i.e., Maslow’s hierarchy of needs) are fundamental to any human livelihood, and certainly for a scientist to be able to flourish. To truly allow independent scientists to develop, a strong set of progressive social policies, such as universal health care, basic income, and high-quality free education, are needed to strengthen the core of the ecosystem ^66, 67^. The ecosystem concept recognizes that the journey of a scientist through training is often an indirect path with many more career development influences than the direct path that a “pipeline” implies ( Figure 1). Instead, an individual learns foundational knowledge, explores ideas, and gathers experience through a journey that is influenced by a broad range of interests, a balance of personal and professional goals, and adaptation to the challenges of life overall.

Such a student might attend the traditional classes expected in their field, explore other fields of interest (e.g., fine arts, social activism), and gather experience through interactions with others, work, internships, and volunteering, both within their field and outside of it. Along the way, they might explore other career (or life) choices, and perhaps return to academia completely, or explore specific scientific questions from a new perspective in another career choice outside of traditional academic institutions. Overall, the ecosystem model emphasizes that ** there is no right way to become a scientist. The diversity of experiences and perspectives are key to advancing STEM development in novel and more inclusive ways.

### 2. Multiplicity of niches

Most importantly, the ecosystem model recognizes that every scientist is a person, meaning that people are more than their jobs and must balance a myriad of responsibilities, goals, and limitations that change as they move through life. While the conventional academic scientist pipeline assumes that individuals are functioning within a protected static environment (i.e., male scientists who serve as the breadwinner for their family in a generally unchanging environment), the ecosystem model encompasses the diverse and dynamic roles that individuals (both men and women) take on, particularly as they move through the household lifecycle (i.e., people’s needs and resources change throughout their life and most notably when they raise a family ^68^). Indeed, sustainable livelihood strategies ^65^ further emphasize this point by recognizing that people must be constantly making decisions to most efficiently use their resources (human, natural, financial, physical, and social capital) to meet their livelihood needs, and such decisions are often made within the context of the changing biophysical and socio-political conditions of the system that they live in.

Furthermore, people must balance both non-monetary activities (i.e., child-rearing, house-keeping) with income earning activities, and both are equally important to almost all individuals. There is a lack of recognition of the importance of non-monetary activities in making livelihood decisions within conventional career models, as well as limited supporting economic and political structures to support these activities ^69^. The pipeline model simply doesn’t consider both career and life balance in such a dynamic environment, nor does it present any opportunities to leverage the social diversity that occurs through such processes. By contrast, the ecosystem model not only presents a flexible model that encompasses the dynamism of the system, it also thrives on social, economic, and experiential diversity.

Income-generating activities in the ecosystem approach can be diverse different, contrasting with the expectation of there being a sole niche, such as tenure-track employment in university settings. Some of these new niches include part-time, or “fractional scholarship” ^57^, through virtual institutes such as the Ronin Institute ^70^ (see Box 2), small business or consulting companies (either partly or wholly focused on scientific research), and non-traditional start-ups. For example, some independent scholars run consultancies involving their scientific expertise in a commercial setting, but reserve time to pursue their own research; their research and consulting activities help inform the other, resulting in more grounded research and science-informed solutions, respectively. At a larger scale, some independent scientists have obtained venture capital funding to pursue biomedical research ^71, 72^, such as Perlara in San Francisco, which operates as a public benefit “B-corporation”.

Box 2. **Independent institutes and laboratories**

Organizations of independent scholars, such as the non-profit Ronin Institute for Independent Scholarship (of which the authors are all members), the National Coalition of Independent Scholars, the Institute for Globally Distributed Open Research and Education, CORES Science and Engineering, Neurolinx Research Institute and research consortia such as the Complex Biological Systems Alliance ^2^ enable highly trained individuals to contribute to basic science outside the traditional academic setting. Independent labs focusing on more specific research questions or subject areas have also emerged, such as the Orthogonal Lab. The Ronin Institute, in particular, provides support and infrastructure for submitting grants to existing funding agencies, but crucially also provides a home for scientists who may have either a part-time or full-time job, but wish to pursue their scholarship on a part-time or “fractional” basis ^57^. Many such scholars also retain joint or visiting status with traditional universities, demonstrating the porousness between institutes as part of the overall scientific ecosystem. Through seminars, virtual meet-ups, and in-person unconferences, the Ronin Institute provides an essential community for independent scholars to trade ideas and identify new collaborations, so that they are not operating within a vacuum. For example, PhD graduates who work in private companies, government agencies, or the non-profit sector do not have to trade their scientific career for a profession. In addition, independent scholars can side-step some of the bureaucracy of the university, while maintaining their scientific identity that can be lost while working in full-time industry jobs.

### 3. Open ecosystem flows

Academics often view the abandonment of the search for a permanent tenured university position as a signal that a person is “leaving science”, but we argue that this should not necessarily be a one-way valve. Thus, another step towards building the open ecosystem is to normalize the movement into and out of traditional university positions. The formal system for scientific training must value students, postdocs, or other researchers who leave and re-enter programs or jobs for their professional and diverse experiences, and the unique network of colleagues that they bring to programs or jobs. Such a change will reduce the fear that scientists have in diversifying their career experience. We expect that for some kinds of science (i.e., those that require the use of expensive equipment), employment in a traditional university-based setting may continue to be the most appropriate, but other types of science (e.g., theoretical and computational sciences) can easily be practiced outside traditional academic settings ( Box 2). Normalizing these movements as one of many flows within the overall scientific ecosystem would be a big step in the right direction ( Box 3) for both broadening and diversifying science, and creating new career opportunities for scientists.

Box 3. **Shifting the dominant narrative**

A tenure-track job is still the dominant yardstick of legitimacy for a scientist ^27^, and such a lack of vision for scientific careers makes institutional and cultural change in science difficult. Benderly (2015) offers one example where non-tenure-track early career scientists have been dismissed in biomedicine ^77^. Unfortunately not all in positions of power are good-faith participants in this conversation: beneficiaries of a system built on implicit assumptions of zero-sum competition for attention and prestige are unlikely to welcome change. However, many senior academics recognize the unsustainability of the current system ^78^. There are many steps that such sympathetic senior academics can take to support the ecosystem view. Here are just some: (i) Use language more carefully: don’t refer to scientists who do not secure a traditional academic job as “leaving the field” ^79^; (ii) More visibly reject the journal impact factor prestige system and embrace preprints and other forms of open research ^80, 81^; and (iii) Include collaborators beyond those who are university-based, when possible. These small shifts will add up, especially if they originate from well-respected senior academics.

### 4. Diversity of scales in pace and budget

The increasingly all-consuming competitive nature of academic life often discourages speculation, innovation, and collaboration ^73– 75^. Little time and energy is left for the reflection needed to develop original ideas ^35^. Interesting and creative science does not necessarily require this intense pace and may even be inhibited by it. The “slow” science movement encapsulates a more deliberative and conscious approach to science ( Box 4). Smaller-scale research projects can have quite modest budgets, and crowdfunding sites such as Kickstarter and Experiment.com have supported many such interesting scientific projects ^76^. Relative to standard grants, funds raised via crowdfunding might be considered tiny, but with the large overhead required by brick and mortar academic institutes (often over 50% of a grant), such funds can often go a long way in a research budget. Such monies have been fairly modest to date, but these approaches are still in their infancy and have much more potential for growth. The scientific ecosystem concept explicitly recognizes the value of, and enables, such scientific work at multiple niches and scales.

Box 4. **The slow science movement**

Today, many grant dollars are awarded to a disproportionately small number of established investigators ^11^, due in part to an increasing emphasis on high-budget, complex science. The rise of “Big Institute” science is channelling both philanthropic and federal dollars towards specific industries (e.g., biomedical research, specific diseases) and biotech hubs ^82^, leading to fewer large labs working in a limited number of lucrative research areas. Traditional labs in university settings are incentivized to grow in size, partly because the overhead earned through grants helps fund the institution as a whole ^42^. “High profile” journals also tend to favor work done at larger, and therefore more expensive, scales. These two factors bend institutional incentives towards funding expensive research questions and hiring faculty with publications in prestigious journals who do such work.

By contrast, the ecosystem model explicitly acknowledges the value of “slower” approaches to scholarship and science, and recognizes that scientific progress is enabled by scientists pursuing questions at all different scales and pace ^83– 85^. While expensive, big science certainly has an important place, its prioritization over smaller-scale investigator-driven science tacitly and severely constrains the scope and types of questions that can be asked ^82, 86, 87^. The slow science movement emphasizes a more deliberative, less publish-or-perish pace to provide time for scientists to build trust, create more effective and durable collaborations, and invest in more collective approaches for doing science. The presence of the collaborative and reflective personalities required to address complex problems with team-based solutions ^88– 91^ tend to be weeded out in the current system. Moreover, even in traditional academic positions, the conventional narrative of faculty publishing productivity—a peak of publications in early career with a gradual decline—doesn’t fit the actual publication records of many scientists. In computer science, for example, it only describes about one-fifth of tenure-track careers ^92^, even though this conventional narrative has a great influence on hiring and tenure decisions.

### 5. Peer-to-peer networks

Researchers in traditional settings operate in a highly hierarchical system, where the usual benchmarks of “success” are controlled by a relatively small number of people at each level. A large number of apprentices are under the control of a small number of masters ^93^; this structure can make it difficult for new ideas to gain a toehold ^94, 95^ and can also lead to the exploitation of apprentices ^96, 97^. While senior investigators occupy important roles in identifying promising research avenues, providing synoptic views and institutional memories of their field, and many are tireless in promoting their trainees and helping them succeed, this success generally assumes continuing within the “pipeline” model. In contrast, we believe that moving towards less hierarchical peer-to-peer ecosystem models, which emphasize more democratic decision-making, cooperativity, and solidarity, will lead to a more dynamic and creative scientific enterprise overall ( Box 5).

Box 5. **Beyond the “principal investigator”: new self-organized collaboration models**

The model of academic science that early career investigators are taught to aspire towards (a “principal investigator” directing a large number of apprentices) is essentially feudal in structure; it is a historical product of the structure of academic institutions. This predominant structure dates roughly from Vannevar Bush’s famous 1945 memo, “Science: the endless frontier” ^104^, and is not intrinsic to the discovery process itself. The cultural movement of increasing equity and access, which is driven by the ubiquitous presence of the Internet, the rise of peer-to-peer, commons-based production ^62, 105, 106^, and crowdsourcing ^107^, challenges this feudal/industrial model of the production of cultural goods ^105, 108^, including science. This looser, self-organized approach to intellectual production is demonstrated by the free, libre, or open-source software (FLOSS) movement (which has produced operating systems like GNU/Linux), as well as Wikipedia ^109^.

Contributors to FLOSS projects also occupy different niches: some developers of open-source software are employed by companies (e.g. Red Hat), others contribute only one or two lines of code as volunteers, and most contribute at levels in between. Some scientific projects are run in similar ways ^110^, and there is natural fit with idea of fractional scholarship introduced in our second pattern on the multiplicity of niches (subheading 2): important scientific contributions can be made at whatever time and energy level that an individual can provide. Open-source projects, and peer-to-peer production more generally, are not without structure ^62^. Projects still develop leadership and lines of authority, and the need for mentorship does not disappear ^111^, but leadership is fluid, often chosen by consensus, and based more on time, energy, and enthusiasm rather than an academic title or pedigree ^112^. Consistent with our ecosystem approach, we imagine a multiplicity of solutions whereby the traditional PI structure is but one model within a larger spectrum of more peer-to-peer structures.

### 6. Fluid communication networks and shared access to resources

The burgeoning open science movement ^15, 98^ is a key enabler of the scientific ecosystem by bringing data, models, and resources out from behind institutional walls. Initiatives, such as the Center for Open Science ^99^ and Sage Bionetworks ^100^, have developed pioneering tools to enable large-scale sharing, and thus mining of open biological data by any interested parties without the need for the original infrastructure that generated the data. Access to shared resources, such as lab space, are also part of this shift ( Box 6). In addition, the citizen science ^101^ and indigenous research ^102^ movements reach out to bright and creative individuals outside the academic system who are eager to contribute skill and time to advance science. The growth of open-access publishing will especially benefit the peer-to-peer model proposed here ( Box 6).

Box 6. **Commons-based access to shared resource and open access**

The DIYbiology movement has championed an extremely low-tech, low-cost approach to experimental molecular biology. The emergence of community labs and commons-based co-working spaces (e.g., manylabs.org) are making work possible outside university settings ^113, 114^. Facilities for genome sequencing ^115^ and “rent-by-the-bench” lab space, such as QB3@953 in San Francisco, CA and LabCentral in Cambridge, MA ^116^, can enable lower-cost, lab-based research that might be completely off the radar from more traditional academic labs or large biotech companies. Mathematical and computationally based research is also now well within the reach of many independent scientists. Cloud-based computing servers offered via Google Cloud and Amazon Web Services ^117, 118^ can be done at a fraction of the cost of running a large university-based high-performance computing (HPC) cluster.

Open-access is another strand in an ecosystem model of science. Notably, open-access information now allows many “researched” communities to finally have access to information about their own communities, and take action from a grassroots position (e.g., members of developing countries are now more empowered to take part in science). However, the “author-pays Article Processing Charges” model of many open-access journals will require some rethinking in the absence of institutional support. The incredible uptake of preprints, especially by younger scientists, as a way around the artificial scarcity of the journal prestige system is an encouraging first step ^119^. The growth of low-cost non-commercial models of publishing based on “platform co-operativism” principles ^120, 121^ that are owned and run by scientists are likely to be more equitable and sustainable in the long-run for scholars than venture-capital backed experiments ^122^ www.scholarlyhub.org;). Open science and open access platforms should be focused on the goal of improving communication, scholarship, and learning, rather than being simply a way to extract commercial value from scientist’s labor ^123, 124^. New conferences like OpenCon and FORCE11 are leading the conversation in this area.

### 7. Broader distribution of resources

Self-funding of research can sometimes be sufficient, since many studies, especially in computational, mathematical, and social science fields cost little beyond the time required. Free of arbitrary institutional expectations of “bringing in grant money”, this can be quite liberating. However, many other kinds of research, especially wet lab biology, can be expensive and labor-intensive. The benefit of joining institutes like the Ronin Institute, allows scholars to apply directly for traditional federal grants (e.g., NIH or NSF) with reduced institutional overhead, leading to more efficient use of money for research. However, existing funding agencies, especially federal, are largely geared to favour the already well-funded or those who are working on whatever scientific questions they may be prioritizing that year. More balance is needed to ensure that scientific questions that are valued by society are also represented. Funding solutions outside of the traditional federal agencies and more distributive approaches, whether federally funded or otherwise, must be considered to help fill this gap ( Box 7).

Box 7. **Rethink funding**

Various federal agencies have mechanisms to compensate for the bias towards large institutions and senior, male scientists. For example, the R15 grant mechanism of the National Institutes of Health is restricted towards institutions that receive less than an overall amount per year. The National Science Foundation has grants specifically for postdocs and early career faculty, and they may withdraw or extend a deadline if gender is not well-represented among the applicants. These programs help to correct bias, but still assume participation in the pipeline model. In addition, the massive amount of bureaucracy involved in the submission and reporting of such grants is a barrier to applicants with minimal grant administration support. Attempts to be more explicitly redistributive, such as a proposed cap of three concurrent R01 grants, have been met with fierce resistance ^125^.

Fundamental rethinking of funding to address the current concentration of resources is needed. Here, we present two ideas. First, we propose the “fail fast” model, which comes from the tech start-up world. In this model, many smaller projects receive 6–12 months of funding to pursue an idea. If the idea works, the next phase receives funding. If the idea does not work, the researchers move on to the next idea. The “fail fast” model would support many smaller groups for a shorter period of time. It also addresses the reluctance of many funding agencies to fund riskier research as well as the need to have nearly all of the proposed work complete before writing a grant to get the funds to do said work. This model is unlikely to support students, however, as they often need 3–4 years of stable funding.

Second, we support experimenting with “collective allocation” models, where each qualified scientist is given a fixed basic grant (somewhat analogous to a “basic income”) and also receives additional funds from other scientists who think they would make good use of the grant money ^126^. Each such scientist would also be required to “pay forward” a fraction of the previous years’ grant money to other scientists in the same manner, thus increasing the overall flow of funds through the scientific community. One benefit is that it reduces the time-consuming and costly bureaucratic infrastructure of the grant review system, while still maintaining the positive influence of peer-review. In addition, there could be rules to minimize “gaming” the system by preventing paying it forward to immediate co-authors ^127^. Related ideas include allocating a portion of funding via lottery ^128^. The new head of Science Europe has expressed interest in trialing these some of these new mechanisms ^129^, indicating a growing frustration with the existing grant process and a willingness to experiment with more radical models.

### 8. Institutional change

While we have emphasized existing grassroots movements and trends, this is not because we do not need reform of our institutional practice of science—we certainly do. However, we believe that social and structural changes are often initiated outside institutions, and these efforts can catalyze internal reforms. That said, many of the design patterns have clear institutional analogs. The aforementioned peer-to-peer approaches discussed under subheading 5 can be implemented within institutions as well, particularly at the level of individual labs, by giving postdocs greater autonomy outside of individual projects or grants ^103^. Some institutions, such as the Santa Fe Institute, have postdocs that are tied directly to the institution, rather than an individual professor. Furthermore, institutions should recognize that researchers work in different styles and at a diversity of scales, as discussed under subheadings 2 and 4, respectively, and avoid monolithic ranking and assessing of scholars by external metrics.

Finally, the ecosystem approach of providing multiple on-ramps and off-ramps to academia (subheading 3) together with distributing resources more broadly (subheading 7), point to a reform of the tenure system itself. The tenure system could be made more fluid, and the benefits of tenure (stability, security) extended to more people, rather than a lucky few. A progressive commons-based economy would be one way to realize this vision ( Box 8). Organizations such as Future of Research, which are leading the way in engaging institutions to encourage open-science practices (subheading 6) and shape a more equitable job and funding landscape (subheading 7), should also be vigorously supported ^58^.

Box 8. **A secure, progressive economic system**

As we noted under subheading 1, basic stability and security is a necessary condition for people to flourish. One of the continuing appeals of university-based, tenure-track positions, especially in the United States, is the job security and health-care benefits promised with tenure. Unfortunately in many industries, including academia, job security is going by the wayside; thus, many new progressive economic models are switching from a focus on job security to a focus on income security, and this takes the form of such initiatives as universal health-care and universal basic income ^66, 67^. With this kind of security in place, the developing flexible “gig economy” in science ^132^ is more likely to fully enable scientists to pursue their work in whichever part of the ecosystem that fits them best, rather than in predefined and allocated roles in the pipeline. The dark side of exploitation and insecurity in much of the existing mainstream cultural and gig economy ^133– 135^ is very real, so we advocate for a truly progressive economic system that protects and creates security for all. The benefits of the new flexible peer-to-peer approaches to work must be widely distributed and not concentrated into a small number of hands ^62, 136^.

## Writing a new cultural narrative

Our paper proposes a fundamental change in the way that scientific training and its professional landscape are viewed. In the spirit of our ecosystem concept, we hope to spark discussion, debate, and action rather than offer a complete “turn-key” solution to the current state of careers in science (if such a thing were ever possible). Increased participation and mobilization of scientists and their partners towards such a concept will ultimately determine how the concept evolves in a bottom-up manner. We particularly hope to reach early-career scientists who are making career decisions and developing professional identities, since they will be most actively involved in co-creating this new ecosystem. We envision that scientists at all levels that are seeking to address the challenges faced by scientific institutions may also find inspiration in this paper to help them catalyze change from the inside out.

Up until this point we have focused on the economic, social, logistic, and professional challenges of a new way of conceiving scientific work and careers. These are critical and necessary steps towards creating a more equitable scientific ecosystem. However, even if we demonstrate economically viable and sustainable ways for more scientists to contribute high quality research outside of traditional institutions, we must still overcome a potentially larger hurdle: the dominant cultural narrative of scientific success based on scarcity, especially of certain kinds of jobs ( Box 3). The set of strategies we propose, while highly disparate and pluralistic in approach, are all characterized by a view that success does not need to be a zero-sum game.

The language we use to frame our new narrative is therefore key to cultural change. As Lakoff and Johnson ^130^ point out, metaphors frame our thinking, as well as shape and constrain the cultural narratives of success. The ecosystem metaphor is our attempt to reframe and expand the discussion of STEM careers and science, beyond what has often become a sterile and arid debate about competition and scarcity within academia, by introducing the concepts of open flows, resilience, diversity, and feed-back loops of ecological systems. To this end, in Table 1, we provide a lexicon that highlights the non-zero-sum and egalitarian aspects of our ecosystem model, illustrating the contrasting language between the pipeline and the ecosystem. We do not assume that the pursuit of prestige or financial rewards will wither away; convivial competition clearly has a proper place in science ^131^ and will always co-exist with cooperation (existing somewhere on the spectrum between the two columns in Table 1). But we strongly believe that making science better is not just about “creating better incentives”, but a collective cultural shift beyond viewing competition and individualistic success as the sole defining feature of science (i.e., the pipeline model).

**Table 1.** Establishing a new cultural lexicon for science: comparing the language emphasized in the pipeline and ecosystem metaphors.

	Pattern	Pipeline metaphor emphasizes	Ecosystem metaphor emphasizes
1	Basic development of scientist	Linear: K-12, grad school, postdoc, “superdoc”, tenure-track “What is your job?”	Multiple pathways, life-long learning, multiple jobs, moving into and out of specific roles/industries “What are you working on?”
2	Career model	Single breadwinner in a static environment: singular focus on productivity for a tenure-track job Standardized career ladders defined by a job title in: academia, industry, NGOs Success defined by job title	Diverse family arrangements: dynamically responding to changing needs Multiplicity of niches not restricted to corporate or academic hierarchies: scientific work and identity that transcends job title Self-defined measures of success
3	Academic positions	One-way valve Independence: defined by securing of Assistant Professor position (financial)	Open ecosystem: flows in and out Independence: claimable at any time (conception and pursuit of your own ideas)
4	Budget and pace	One-size fits all, bigger and faster always better “All-or-nothing”: singular focus of life	Diversity of scales, both in pace and budgets “Fractional” science/scholarship
5	Working style	Principal investigator + apprentices Hierarchical, top-down, permissions culture Individualistic, competitive	Peers + collaborators Peer-to-peer, collaborative, permissionless culture Solidarity, cooperative
6	Resource access and publishing models	Private or institutionally based, closed to outsiders Closed-access “high prestige” journals, data hoarding for competitive advantage	Commons-based access: community labs, MakerSpaces, DIY Biology Open science, open access, preprints, data sharing
7	Funding	Competitive, winner-take-all. Concentration of resources in high prestige institutions	Collective allocation, experiment with alternative means of proposal evaluation Wider distribution, not dependent on affiliation.
8	Institutional changes	Keep structure: limit access, train fewer PhDs Scarcity, long-term permanent institutional employment accessible to lucky few	Transform institutions: engage ever more scientists Abundance, platform cooperativism, project-oriented work, basic income, universal health care

The existing socialization process of traditional academic science vastly over values signals of academic capital (title, rank, and institutional affiliation) and economic metrics of productivity (amount of grant funding, high-impact papers, and *h*-index); scholars who do not meet these criteria are often disregarded, limiting diversity in science. The most important part of the shift toward an ecosystem model is cultural and psychological: the essential spirit of science must be re-captured by emphasizing that there are no gatekeepers to the scientific development of knowledge. Academia doesn't “own” science, any more than museums, art schools, or galleries own art. Just as with visual or performing arts or music, the open-ended exploration of scientific ideas is something worth celebrating in itself, regardless of the nature or scale of the question.

Changing the cultures of research careers and the scientific enterprise is an experiment itself: actively practicing new a scientific culture can encourage others to be even bolder in their experimentation. The existing institutions that are tasked with supporting basic curiosity-driven inquiry need to be reformed and strengthened, but that alone is insufficient. We must build new structures that are informed by an ecosystem view from conception. The beauty is that science can be made available to everyone and our technologies are making it increasingly so. It is not a scarce resource: we should build our new ecosystem to recognize this truth.

## Data availability

No data is associated with this article.
