# Supplementary material for: A new paradigm for the scientific enterprise: nurturing the ecosystem
Source: F1000Res. 2018 Jun 20;7:803. [Version 1] doi: 10.12688/f1000research.15078.1 (PMC6171719; doi:10.12688/f1000research.15078.1)
Supplement: Peer review history

# Peer review: Referee response for version 1

**DOI:** 10.5256/f1000research.16419.r38436

**Published:** 2018-10-01

**Authors:** Cameron Neylon

**Affiliations:** 1 Centre for Culture and Technology, Curtin University, Perth, WA, Australia

This article offers a way of thinking about the environment of career structures in science. The other two reviewers have noted a series of small issues which I concur would be good to deal with. The paper overall offers some good critical thinking and summary of issues with the way we think about career structures and job roles and provides advocacy for new ways of thinking. It is generally well written and well argued.

 I am going to raise two broader issues which I think should be addressed before finalising for indexing:

1. Is the 'ecosystem' concept well defined and expressed?
2. Is this a 'new paradigm'? 

There has been much criticism of ecosystem as a metaphor, primarily from ecologists who object that the term is used imprecisely. I think here there is a risk of getting muddled. Words like 'ecosystem' and 'landscape' are used in opposition to the traditional concept of a 'pipeline' but are not explicitly defined or drawn as analogies to true ecosystems.

 I was left wondering whether the 'ecosystem' referred to an ecology of job roles, of workers within those roles, organisations that support them or something else? Figure 2 to me does not evoke an ecosystem but a set of roles and organisations. Rather some elements seem to be drawn from Ostrom's Institutional Analysis and Design model, along with specific instances of change, then roles then knowledge (from outside to in). From a different angle it also looks like a social learning model (moving from centre outwards, Lave and Wenger, Situated Learning). In passing I note a tension between the claim the circle 'has no top' with the line above the figure that '....bottom-up changes are already being driven by early career scientists.

 The model/figure also does not seem to be used further in the examples below. I think this is a sign that the model and narrative is not quite right. If I understand correctly the goal is to use the ecosystem metaphor to emphasise interdependence of *actors* and the ability to work effectively together (adopting from commons language throughout). However the pipeline model refers to *development* of those actors. My suggestion would be to consider more explicitly how the pipeline model presumes hierachical institutional forms and how the new model (community/social learning in communities of practice?) would support new, networked and flexible institutional forms.

 This happens towards the end in 'Writing a new cultural narrative' to a certain extent but I would like to see this made much stronger. I think the relationship between rhetoric, narrative, social learning/acculturation and institutional forms could be made much earlier, ideally in 'The science ecosystem' as an answer to the deficiencies in the pipeline model that are ably described prior to this. This could draw on a range of previous work.

 This brings me to the second point. The claim is made that this is a new model. I am not convinced this is accurate. Elements of the ecosystem metaphor, or at least the division of labour and necessary organisation to create value, are found in Merton (many of the papers in Sociology of Science), Lave and Wenger (Situated Learning), Ravetz (Scientific Knowledge and its Social Problems), Latour (Politics of Nature) and in others discussing the broad area of Science and Technology Studies (Collins on expertise, Stilgoe on public policy development, Stirling on engagement and diversity etc etc).

 More directly in STEM debates around the formation and solidification of Bioinformatics as a discipline involving new technical workers and skills (and more recently Digital Humanities - I think it would be good for the application of the model to humanities and social sciences to be at least gestured at), the formation of research institutes, and massive authorship in astronomy, high energy physics, and some areas of biology have all pointed in the direction of an 'ecosystem' of interdependent roles to support modern large-scale science.

 My primary suggestion is that referring to this earlier literature will strengthen this paper and aid in the development of a stronger narrative, which will in turn hopefully improve the impact of the article in achieving the authors' goals. I would consider re-working Figures 1 and 2:

1. Figure 1 emphasises that the rhetoric of a pipeline leads to a hierarchical structuring of institutions and a necessary conservatism as a result. You might consider adding an 'average age' of each transition, job titles etc to emphasise this. This can then be picked up more strongly in Box 1 as to how the presumed path and hierarchy leads to the negative results described.
2. In Figure 2 I'd invert the sense of the diagram. Start at the outside and show many spiral paths inwards to a range of roles and organisations (can the relationship between organisations and roles be shown). This echoes Lave and Wenger's concept of learning as 'legitimate peripheral participation'. It then becomes important to emphasise that there are many such target diagrams and that various paths can encompass several of them.

I think these two modifications make it easier to refer back to the figures in the examples and cases described in the numbered sections and boxes. That in turn will make the whole narrative stronger.

I have read this submission. I believe that I have an appropriate level of expertise to confirm that it is of an acceptable scientific standard, however I have significant reservations, as outlined above.

# Peer review: Referee response for version 1

**DOI:** 10.5256/f1000research.16419.r35293

**Published:** 2018-09-21

**Authors:** Gary S. McDowell

**Affiliations:** 1 The Future of Research Inc., 848 Brockton Avenue, Abington, MA, USA

Overall this is an extremely interesting thesis and lays out a lot of the current issues with “the pipeline” succinctly. I have made a number of suggestions that I hope the authors find interesting or useful in revising the paper, and a request for citations. I have marked the paper as approved but am hopeful the authors are receptive to revising based on comments below.

 Major comments:

 I would clarify when you are talking about a) biomedicine vs all fields and b) issues in the U.S. vs issues globally, where possible. For example, in the introduction you state: “Declining public funding for basic science” and cite examples that are particularly focused on biomedicine in the U.S. Checking throughout the manuscript to ensure that geographical/field limitations of data cited are highlighted and differentiated would be very helpful to see where issues can be generalized or not, and may led to further interesting insights where differences do or do not arise. While the biomedical enterprise in the U.S. is certainly useful as the “canary in the coalmine”, it would be interesting to set this in context.

 I would suggest looking into the work of Dr. Wayne Wahls ^1^^-^^2^ and Dr. Kenneth Gibbs Jr. ^3^^-^^7^ and also Levecque et al. 2017 ^8^ and Piefer 2017 ^9^ and considering citing/including any reactions to these in your discussions.

 In particular the work of Dr. Gibbs may also help inform a discussion throughout of diversity in terms of gender and race/ethnicity; a key strength of the ideas proposed in this paper is that it can tie into making an enterprise that would help people align their interests with the reasons to stay in various parts of the scientific ecosystem, and in particular diversifying academic faculty through a more fluid scientific ecosystem would be very interesting to explore here.

 Minor comments:

 Introduction: 

 Suggest deleting “which are” in last sentence of third paragraph in introduction.

 “In 2016 $3 billion were invested into federal agencies to support STEM education programs” - another interesting number would be an estimated amount that is spent by the taxpayer on training writ broadly - i.e. not just training mechanisms and education programs, but also considering that 85% of grads and postdocs funded by NIH (including all foreigners) are not funded from NRSA training mechanisms. Or could it be approximated what proportion of NIH’s budget is linked to trainees in some way? It may also be worth mentioning the previous interest Congressionally in the sustainability of the training enterprise, which led to the creation of the NRSA (see p6 in Addressing the Nation's Changing Needs Report ^10^) - which contains, again, only 15% of its trainees.

 Box 1:

 “Postdoctoral training periods also continue to expand” - please provide a citation, or points of comparison i.e. specific lengths in specific years, also again specifying countr(y/ies) and field(s). One place to look for data that addresses that talking points may be the recent National Postdoc Survey ^11^.

 In the discussion of the default faculty path it might be useful to include Dr. Paula Stephan’s analysis of NSF data showing the proportions of U.S. biological sciences PhDs entering particular job sectors 5 years post-PhD (only published in The Atlantic in https://www.theatlantic.com/business/archive/2013/02/the-phd-bust-americas-awful-market-for-young-scientists-in-7-charts/273339/) and also Figure 2-1 in the NASEM “Breaking Through” report could be of interest too ^12^. Dr. Stephan’s analysis in particular shows that tenure-track faculty positions have not been the principal (let alone majority) destination for PhDs for the lifetime of most Millenials, which allows you to qualify how outdated this mentality is.

 “unencumbered with children” - it may be worth looking further into this - in our analysis with the U.S. Census Bureau in http://sjscience.org/article?id=570 and the related Nature Comment piece ^13^, The New Face of US science - the latter in particular points out specifically that men in their 30s are 7x more likely to have a non-working spouse, and many who are married tend to have children, and in fact there may be some discussion to be had about whether being married is beneficial. Men are more likely to be married than women, but women are more likely than men to be married to someone who is *also in the biomedical workforce*. Combining these factors with the advantage that tenure clock interventions designed for women actually confer on men ( http://sjscience.org/article?id=570) it may be that the “unencumbered with children” comment requires qualifying, or at least some rethinking/citations to show that being childless does confer an advantage. Again the National Postdoc Survey could be useful to check into here.

 Figure 1: I would suggest with industry also breaking out not only into academic/non-academic but also research/non-research. In preparing the National Academies report, Breaking Through you see in Figure 2-1 a representation of the phenomenon that many industry jobs are not bench-based, but are sales or managerial positions. Consultancy should also be a large group. This would be useful to illustrate the need for the ecosystem even more, as even “industry” is complicated. It might also be worth looking at the taxonomy work that has been going on nationally ^14^.

 Fixing the pipeline:

 I was somewhat confused by the angle the authors were coming from in the last sentence in the “flow in the pipeline” discussion. Why would fewer scientists with more career stability be a bad thing in the tenure-track path? Are they proposing instead more scientists with more career stability, in a diverse range of careers? The authors should be careful not to fall into the “more scientists are better for society” argument, as data about the labor market and PhD outcomes is scarce but points to a massive gap in the labor market with supply far exceeding demand, across all jobs ^15^. If however they are trying to make the argument that fixing flow is too restrictive a view/the issues that arise in focusing only on flow, I believe all but the last sentence support this view. Some expansion here to clarify what is meant would be helpful. Having read through the rest of the Fixing the Pipeline section I am satisfied that clarification is all that is needed here, because I get a strong sense of the line of argument in the other sections.

 The adapting to the pipeline argument is very well made. It could also be worth pointing out that this is causing pressure to extend the length of postdoc positions, because it is taking longer to get a position, rather than changing the system so that postdoc positions are not so long i.e. by evaluating metrics other than high IF publications in competition with other applicants. I am glad to see the argument being made about for-profit services, it might be worth considering also asking why institutions are not providing such services when they receive federal funds to train these trainees.

 In the thinking outside the pipeline section it could also be worth pointing out the lack of access to alumni networks, or the poor communication between academics and non-academics, and also the simple inability of mentors, no matter how well-meaning, to advise on careers they themselves do not know about. It is also worth considering that many trainees do not feel comfortable bringing up that they may wish to leave academia, even though the PI may be receptive to this idea. This is largely due to over-arching cultural expectations, which also include the idea that “to leave is to fail”, which could also be mentioned somewhere in here.

 Box 3 - It may also be worth suggesting a vital role professional societies can play in ensuring that “the field” is not synonymous with academia.

 There was a lot of excellent content in the description of the scientific ecosystem: I would suggest considering a way of placing key elements of this discussion in a figure, as there was a lot of information, and it was sometimes difficult to appreciate all the points being made. Some way of parsing out the information in a slightly more digestible format would be very helpful, to really draw in readers and highlight some of the excellent points made.

I have read this submission. I believe that I have an appropriate level of expertise to confirm that it is of an acceptable scientific standard.

## References

[1] WahlsW: High cost of bias: Diminishing marginal returns on NIH grant funding to institutions. *bioRxiv*.2018; 10.1101/367847 10.1101/367847

[2] WahlsWP: Biases in grant proposal success rates, funding rates and award sizes affect the geographical distribution of funding for biomedical research. *PeerJ*.2016;4: 10.7717/peerj.1917 e1917 10.7717/peerj.1917 27077009PMC4830231

[3] GibbsKBassonJXieraliIBroniatowskiD: Decoupling of the minority PhD talent pool and assistant professor hiring in medical school basic science departments in the US. *eLife*.2016;5: 10.7554/eLife.21393 10.7554/eLife.21393 PMC515324627852433

[4] GibbsKMarstellerP: Broadening Participation in the Life Sciences: Current Landscape and Future Directions. *CBE—Life Sciences Education*.2016;15(3) : 10.1187/cbe.16-06-0198 10.1187/cbe.16-06-0198 PMC500889827587849

[5] GibbsKDMcGreadyJGriffinK: Career Development among American Biomedical Postdocs. *CBE Life Sci Educ*.2015;14(4) : 10.1187/cbe.15-03-0075 ar44 10.1187/cbe.15-03-0075 26582238PMC4710405

[6] GibbsKDMcGreadyJBennettJCGriffinK: Biomedical Science Ph.D. Career Interest Patterns by Race/Ethnicity and Gender. *PLoS One*.2014;9(12) : 10.1371/journal.pone.0114736 e114736 10.1371/journal.pone.0114736 25493425PMC4262437

[7] GibbsKDGriffinKA: What do I want to be with my PhD? The roles of personal values and structural dynamics in shaping the career interests of recent biomedical science PhD graduates. *CBE Life Sci Educ*.2013;12(4) : 10.1187/cbe.13-02-0021 711-23 10.1187/cbe.13-02-0021 24297297PMC3846521

[8] LevecqueKAnseelFDe BeuckelaerAVan der HeydenJGisleL: Work organization and mental health problems in PhD students. *Research Policy*.2017;46(4) : 10.1016/j.respol.2017.02.008 868-879 10.1016/j.respol.2017.02.008

[9] PeiferM: The argument for diversifying the NIH grant portfolio. *Molecular Biology of the Cell*.2017;28(22) : 10.1091/mbc.e17-07-0462 2935-2940 10.1091/mbc.e17-07-0462 PMC566225329084912

[10] National Research Council US Committee on National Needs for Biomedical and Behavioral Scientists: Addressing the Nation's Changing Needs for Biomedical and Behavioral Scientists.2000; 24967499

[11] McConnellSWestermanEPierreJHecklerESchwartzN: Career Choice, Gender, and Mentor Impact: Results of the U.S. National Postdoc Survey. *bioRxiv*.2018; 10.1101/355511 10.1101/355511 PMC629878330561332

[12] Committee on the Next Generation InitiativeBoard on Higher Education and WorkforcePolicy and Global AffairsNational Academies of Sciences, Engineering, and Medicine: The Next Generation of Biomedical and Behavioral Sciences Researchers.2018; 10.17226/25008 10.17226/25008

[13] HeggenessMGunsalusKPacasJMcDowellG: The new face of US science. *Nature*.2017;541(7635) : 10.1038/541021a 21-23 10.1038/541021a 28054625

[14] StayartCBrandtPBrownAHuttoTLaytonRPetrieKFlores-KimEPeñaCFuhrmannCMonsalveG: Applying Inter-rater Reliability to Improve Consistency in Classifying PhD Career Outcomes. *bioRxiv*.2018; 10.1101/370783 10.1101/370783 PMC701458032089837

[15] MasonJLJohnstonEBerndtSSegalKLeiMWiestJS: Labor and skills gap analysis of the biomedical research workforce. *FASEB J*.30(8) : 10.1096/fj.201500067R 2673-83 10.1096/fj.201500067R 27075242PMC4970602

# Peer review: Referee response for version 1

**DOI:** 10.5256/f1000research.16419.r35295

**Published:** 2018-06-25

**Authors:** Jonathan P. Tennant

**Affiliations:** 1 Open Science MOOC, Berlin, Germany

Herein is my response to the manuscript entitled “A new paradigm for the scientific enterprise: nurturing the ecosystem”, by Alexander Lancaster and colleagues to F1000 Research.

 The authors present a criticism of the concept of the ‘academic pipeline’, and instead introduce a new ecosystem model that includes more dimensions and scope for understanding the complexity of academic culture. My relevant ‘expertise’ in reviewing this manuscript comes from being a researcher interested in developments in scholarly communication. As this is an opinion piece, the only real substance I can offer is that of my own opinion, as well as attempt to help improve the clarity and argumentation style of the article. I hope the authors find the comments useful and know that this was an enjoyable and provocative read for me.

**Figures**

 Figure 1. The arrows in the green academia arrow seem to be misaligned. Yes, I am making this comment. Do you by any chance have statistics that could help to quantify this flow diagram? I know the Royal Society have a very similar one for the UK, but would be cool (but not obligatory) to get some numbers on this. Great figure though, and very easy to interpret.

 Figure 2. Given that this is an open peer review, it should perhaps come as no surprise that I think Open Access should be changed to Open Science. So I found this a little annoying flicking between the caption and the image to interpret it. Is it possible to put a very simply coloured legend on the side as well to help maintain focus?

**Abstract**

 The abstract is concise, interesting and impactful, and onveys the context and main findings of the article.

**Introduction**

 The first paragraph of the Introduction is basically identical to the Abstract, which seems a bit odd to me. Can one be rephrased, or is there a different way of introducing the issue?

 Second paragraph, can early career be defined. And does every country use dollars? Here, I think it would be worth mentioning that it is not just a funding issue, but that this has been exacerbated by the incentive/reward system, which has become virtually divorced from the issues raised here (i.e., anything vaguely scientific). The final sentence of this paragraph needs a couple of citations to support too, or to be stitched to the beginning of the following paragraph.

 Third paragraph. Do you think there are phases between training and permanent positions? I’m thinking of short-term contract postdocs, for example.

 Box 1. Can it be mentioned here that, as a PhD student/ECR, you are often almost guilted into this state, as leaving for a job outside of academia is perceived as ‘failure’ by those within? As well as this, many bright young students come into this system, full of so much potential and energy, and then become disillusioned by it and leave as a result. Which basically creates a bias in which it is those who are conformist, the privileged, or have more support (or just a stronger desire) who end up staying. In the paragraph on families, I would make it clear that this present state is “actively discriminating” against those who cannot conform to that lifestyle. True, and impactful. The content of this box though is very well written and conveys many of the large issues in academia very well.

 Paragraph 5. What is the fundamental cultural change?

 The introduction is well-written, provides sufficient context and background for the piece, and finishes with a nice summary of the work within.

**Fixing the pipeline**

 First paragraph. Should make it clear here, I think, that by framing it as a pipeline it constrains the potential scope for solutions.

 Adjusting the flow. I think it is imperative to make the point here that adjustments at this end do not impact all demographics equally. For example, by raising costs to reduce student numbers, this discriminates against different groups based on their financial status. Here, could one or two specific examples of changes to the supply side be provided, and what their impact was.

 Adapting to the pipeline. Here, I think a comment is needed about the divergence between what it means to be a good academic in order to conform to the system and advance one’s career, and the fundamentals of research. For example, this has repercussions on how research is performed and communicated.

 Finding another pipeline. In the final sentence, do you think this goes wider than just science though, and is an outcome of an increasingly neoliberal academy?

 Thinking outside the pipeline. So, once you’re ‘in the pipeline’, is it very difficult to see outside the pipeline, do you think? And do you think reform is so slow because we are waiting for it to come from the top down? “I made it here, so why can’t you. Nothing needs to change.”

**The science ecosystem**

 Personal preference, but I would love it if somewhere here the UN Sustainable Development Goals could be mentioned ( https://sustainabledevelopment.un.org/).

 Do you feel that, within the rapidly developing Open Science ‘movement’, researchers are being taught the core skills and competencies they need to maintain their careers? See the EU report on this from 2017: https://publications.europa.eu/en/publication-detail/-/publication/3b4e1847-c9ca-11e7-8e69-01aa75ed71a1/language-en.

 Fundamental development of the scientist. For me, journey implies pathway, which is kind of similar to a pipeline. I think the key here is that development is part of an immensely complex network, where every interaction, every thought, every event has some sort of untraceable impact. You could say, to use your own analogy, that each individual has their own ecological niche within a hierarchical network, and that niche is defined by an incredibly multi-dimensional series of developmental traits. I like this analogy. Does an increased diversity of developmental traits decrease the chance of extinction?

 Multiplicity of niches. A key here is the recognition that an individual is the product of their ecosystem. Therefore, it takes into account developmental history (point 1), as well as external factors over which they have no control. As well as genetic heritability, which I think in academia is manifested as privilege. Is there any data out there on the proportion of researchers who have secondary jobs or income-generating activities? Thinking things like teaching, writing, public talks etc.

 Open ecosystem flows. As someone who is independently pursuing research beyond academia, I think that this section is critical. Academia is vastly prohibitive to numerous dimensions of social mobility, and allowing greater physical flow is also important for researchers to take advantage of the power of networked worlds. Research does not need always need a desk.

 Box 3: As well as what is already mentioned, how about support fairer research evaluation initiatives like DORA ( https://sfdora.org/), as well as organisations like Future of Research ( http://futureofresearch.org/). There could be whole papers written about this, so appreciate if the authors don’t want to list all possibilities here!

 Diversity of scales and budget. I would mention here the impact that this can have on research. Salami slicing for publications, claims of data ownership and generally selfish attitudes, data doctoring and manipulation, fraud, other questionable research practices – all driven by hyper-competition and the modes of research evaluation. Box 4 might be better for this though.

 Box 4. ““High profile” journals also tend to favor work done at larger, and therefore more expensive, scales.” – citation needed.

 Peer to peer networks. It’s a shame the way the present power dynamic works. Even if such a research apprentice should be fortunate enough to sit on a high-level council, they might not be granted the rank of master. This can have disastrous consequences for the Galaxy.

 Box 5. I like this idea of sort of decentralised power structures within academia. Are there any examples you can think of where Wiki- or FLOSS-like communities exist within research specifically? A recent study by Hartgerink and van Zelst ^1^ on peer to peer networks might be interesting to mention here too. What impact do these approaches have on inclusivity, as well as prestige or reputation for belonging to such a community?

 Fluid communication networks and shared access to resources. So, this is about exposing just the outputs of research, then? What about wider engagement in science, especially from citizens? And more transparency into the processes, to help enable verification, trust, and collaboration in research? Note that this aspect also includes factors such as open evaluation, and therefore incentive structures within academia.

 Box 6. Should probably mention something about the principles of the scholarly commons ( https://zenodo.org/record/569952#.Wy0XHIjRBPY, and other links). I would perhaps mention some of the causes for this open science movement, such as negative feelings towards the publishing industry, non-scientific evaluation criteria, reproducibility issues, a demand for greater transparency and public accountability etc. If you really wanted, you could discuss some of the wider impacts of Open Access too ^2^. Might also be worth mentioning that commercial publishers are rapidly moving into this area too, such as the recent launch of the EU Open Access Monitor, which was sub-contracted to Elsevier, one of the scholarly publishers with the worst reputation (which I’m sure I don’t have to tell you; https://ec.europa.eu/info/research-and-innovation/strategy/goals-research-and-innovation-policy/open-science/open-science-monitor/about-open-science-monitor_en).

 Broader distribution of resources. How many researchers does Ronin currently support? And how do we work out which questions are more valued by society?

 Institutional change. How about institutes recognizing different forms of scholarship, beyond just research papers? So, training, software, code, tech support, educational resources, among other things. I think that it’s important to mention the link between institutional change and incentives.

**Writing a new cultural narrative**

 This sums up the content of the work nicely, and makes it clear what the intention of it was. I wonder, just to strengthen it, could you perhaps provide just several simple, concrete steps that the different stakeholder groups can take towards reaching such a new culture? Otherwise, I find this section very inspirational!

 Congratulations to the authors on a great piece of work. I think that this could be very impactful and spark substantial new discussion and action in this area.

I have read this submission. I believe that I have an appropriate level of expertise to confirm that it is of an acceptable scientific standard.

## References

[1] HartgerinkCvan ZelstM: “As-You-Go” Instead of “After-the-Fact”: A Network Approach to Scholarly Communication and Evaluation. *Publications*.2018;6(2) : 10.3390/publications6020021 10.3390/publications6020021

[2] TennantJPWaldnerFJacquesDCMasuzzoPCollisterLBHartgerinkCH: The academic, economic and societal impacts of Open Access: an evidence-based review. *F1000Res*.2016;5: 10.12688/f1000research.8460.3 632 10.12688/f1000research.8460.3 27158456PMC4837983
